# Defining nosocomial transmission of *Escherichia coli* and antimicrobial resistance genes: a genomic surveillance study

**DOI:** 10.1016/S2666-5247(21)00117-8

**Published:** 2021-07-05

**Authors:** Catherine Ludden, Francesc Coll, Theodore Gouliouris, Olivier Restif, Beth Blane, Grace A Blackwell, Narender Kumar, Plamena Naydenova, Charles Crawley, Nicholas M Brown, Julian Parkhill, Sharon J Peacock

**Affiliations:** Department of Infection Biology, Faculty of Infectious and Tropical Diseases, London School of Hygiene & Tropical Medicine, London, UK; Department of Medicine, University of Cambridge, Cambridge, UK; Department of Infection Biology, Faculty of Infectious and Tropical Diseases, London School of Hygiene & Tropical Medicine, London, UK; Department of Medicine, University of Cambridge, Cambridge, UK; Cambridge University Hospitals NHS Foundation Trust, Cambridge, UK; Department of Veterinary Medicine, University of Cambridge, Cambridge, UK; Department of Medicine, University of Cambridge, Cambridge, UK; EMBL-EBI, Wellcome Trust Sanger Institute, Hinxton, UK; Department of Medicine, University of Cambridge, Cambridge, UK; Cambridge University Hospitals NHS Foundation Trust, Cambridge, UK; Cambridge University Hospitals NHS Foundation Trust, Cambridge, UK; Public Health England, London, UK; Department of Veterinary Medicine, University of Cambridge, Cambridge, UK; Department of Medicine, University of Cambridge, Cambridge, UK; Cambridge University Hospitals NHS Foundation Trust, Cambridge, UK

## Abstract

**Background:**

*Escherichia coli* is a leading cause of bloodstream infections. Developing interventions to reduce *E coli* infections requires an understanding of the frequency of nosocomial transmission, but the available evidence is scarce. We aimed to detect and characterise transmission of *E coli* and associated plasmids in a hospital setting.

**Methods:**

In this prospective observational cohort study, patients were admitted to two adult haematology wards at the Cambridge University Hospitals NHS Foundation Trust in England. Patients aged 16 years and older who were treated for haematological malignancies were included. Stool samples were collected from study participants on admission, once per week, and at discharge. We sequenced multiple *E coli* isolates (both extended spectrum β-lactamase [ESBL]-producing and non-ESBL-producing) from each stool sample. A genetic threshold to infer *E coli* transmission was defined by maximum within-host single nucleotide polymorphism (SNP) diversity and the probability of drawing observed pairs of between-patient isolates at different SNP thresholds. Putative transmission clusters were identified when sequences were less than the genetic threshold. Epidemiological links for each transmission event were investigated. We sequenced all *E coli* positive blood samples from the two adult haematology wards.

**Findings:**

We recruited 174 (51%) of 338 adult patients admitted to the wards between May 13 and Nov 13, 2015. We obtained and cultured 376 stool samples from 149 patients, of which 152 samples from 97 (65%) patients grew *E coli*. Whole-genome sequencing was done on 970 isolates. We identified extensive diversity in the bacterial population (90 sequence types) and mixed *E coli* sequence type carriage. 24 (26%) patients carried two sequence types, 12 (13%) carried three, and six (6%) patients carried four or more sequence types. Using a 17 SNP cutoff we identified ten clusters in 20 patients. The largest cluster contained seven patients, whereas four patients were included in multiple clusters. Strong epidemiological links were found between patients in seven clusters. 17 (11%) of 149 patients had stool samples positive for ESBL-producing *E coli*, the most common of which was associated with *bla*_CTX-M-15_ (12 [71%] of 17). Long-read sequencing revealed that *bla_CTX-M-15_* was often integrated into the chromosome, with little evidence for plasmid transmission. Seven patients developed *E coli* bloodstream infection, four with identical strains to those in their stool; two of these had documented nosocomial acquisition.

**Interpretation:**

We provide evidence of bacterial transmission and endogenous infection during routine care by integrating genomic and epidemiological data and by determining a genetic cutoff informed by within-host diversity in the studied population. Our findings challenge single colony-based investigations, and the widely accepted notion of plasmid spread.

## Introduction

*Escherichia coli* is one of the leading causes of bloodstream and urinary tract infections, a proportion of which are health-care associated.^1^ Rates of *E coli* bloodstream infections have markedly increased in numerous countries, such as England, where the incidence rose from 60·4 per 100 000 population (32 309 reported cases) in 2012–13, to 77·3 per 100 000 population (43 209 reported cases) in 2019–20.^1^ This rate has increased from 76·6 to 121·6 per 100 000 population at Cambridge University Hospitals in England within the same period.^2^ This problem is compounded by a global increase in the frequency of *E coli* infections caused by strains that are resistant to numerous antibiotics, which are associated with excess morbidity, mortality, longer hospital stays, and higher health-care costs.^3–6^

Interventions to support a reduction in health-care associated bloodstream infections caused by *E coli* require an understanding of the frequency of nosocomial transmission, but the available evidence remains scarce. Previous studies^7–9^ that used bacterial sequencing, an essential tool that provides the necessary genetic resolution, were done on small cohorts or included solely extended spectrum β-lactamase (ESBL)-producing *E coli* or specific sequence types, which were more likely to under-represent transmission of *E coli* overall (ie, including both ESBL-producing and non-ESBL-producing *E coli)*. Furthermore, transmission studies require an understanding of the frequency of mixed strain *E coli* carriage and within-host diversity of the same lineage.

The aim of this study was to report findings from genomic surveillance of *E coli* in two haematology hospital wards.

## Methods

### Study design and participants

We did a prospective observational cohort study of patients admitted to two adult haematology wards at the Cambridge University Hospitals NHS Foundation Trust in England. We evaluated *E coli* acquisition and transmission in patients aged 16 years and older, who were treated for haematological malignancies. Patients younger than 16 years and those not treated for haematological malignancies were excluded. All patients provided written informed consent. The same cohort was previously studied to investigate the transmission of *Klebsiella pneumoniae* and *Enterococcus faecium.^10,11^* The study protocol (appendix 1 pp 2–6) was approved by the National Research Ethics Service (14/EE1123 and 12/EE/0439) and the Cambridge University Hospitals NHS Foundation Trust (A093285 and A092685).

### Procedures

Hospital admission and bed movement data were extracted electronically using the hospital bed tracking system. Admission to the same bay, room, or ward at the same time or within 7 days was classified as a strong epidemiological link; admission in the same ward separated by more than 7 days or to the study hospital but to different wards (regardless of admission dates) was classified as a weak epidemiological link; and no epidemiological link was reported if neither of these occurred. After enrolment, patients provided stool samples during admission and then once a week until discharge. Samples were enriched in Tryptic Soy Broth (Sigma, Dorset, UK) and directly cultured onto Brilliance UTI Chromagar (Oxoid, Basingstoke, UK) to detect all *E coli* and onto Brilliance ESBL agar (Oxoid, Basingstoke, UK) to detect ESBL *E coli*. Up to 15 *E coli* colonies (ten putative ESBL-producing and five non-ESBL-producing), cultured from each stool sample, were selected for sequencing (appendix 1 p 2). For stool samples that grew fewer than 15 *E coli* colonies, all of the available colonies were sequenced.

During the 6-month study, any blood cultures taken from the patient cohort based on clinical need were identified in the diagnostic laboratory. If the sample grew *E coli*, the primary subculture plate was identified and retrieved from the diagnostic laboratory and up to 12 colonies were picked for sequencing. Hospital acquired and health-care associated infections were based on definitions by Friedman and colleagues.^12^ Additionally, we retrospectively identified all blood cultures positive for *E coli* in patients residing in the two haematology wards in the 12 months before this study (between May 13, 2014, and May 13, 2015) and 6 months after (between Nov 13, 2015, and May 13, 2016), from which one colony was obtained for sequencing from the culture in the freezer archive. Further details on culture protocols, selection of colonies, and antimicrobial susceptibility testing are shown in appendix 1 (p 2). The number of invasive infections per 1000 admissions was determined from the number of recruited patients admitted to haematology wards.

The objectives of this study were to measure within-host *E coli* diversity, identify potential clusters of *E coli* transmission between patients at the study sites, and identify associated plasmids encoding antimicrobial resistance genes in this setting.

### Sequencing and bioinformatic analyses

DNA was extracted, libraries prepared, and sequenced on an Illumina HiSeq2000 (San Diego, CA, USA) with 125 cycle paired-end reads. Following quality control, genomes were assembled using SPAdes (version 3.11.0) and mapped against the *E coli* reference strain (GenBank: LT632320) using SMALT (version 0.7.4).^13^ In-silico sequence type identification of all sequenced isolates was done using the EnteroBase MLST sequence archive. Sequencing and bioinformatic methods are described in appendix 1 (p 3). The core genome was derived using Roary (version 1.7.1) using the “don’t split paralogs” option.^14^ Whole-genome alignments were created by calling nucleotide alleles along the *E coli* LT632320 reference genome and pairwise single nucleotide polymorphism (SNP) distances in core genome alignments using pairsnp (appendix 1 pp 3–4). The core genome coordinates are publicly available. SNP distances cannot be compared with whole-genome SNP differences, but should be comparable with the distances reported using the same reference genome and coordinates used in this study. The genomes of multiple *E coli* isolates from the same patient were used to ascertain *E coli* within-host diversity for all participants and subsequently determine an appropriate threshold to define transmission of *E coli* sequence types between patients. The analysis was limited to instances in which different patients shared the same sequence type. The upper limit for a SNP cutoff was provisionally established from the maximum within-host diversity (the number of core genome differences in isolates of the same sequence type from the same patient), which defines the upper limit of transferable diversity from one person to another. The SNP cutoff was validated using a statistical approach, as described in appendix 1 (pp 4−5). The cutoff was applied to all pairs of sequences to identify putative transmission and was subsequently complemented with epidemiological information.

### Detection of antimicrobial resistance and mobile elements

Antimicrobial susceptibility testing was determined for all isolates using the N206 card on the Vitek 2 instrument (bioMérieux, Marcy l’Étoile, France), calibrated against EUCAST breakpoints. Detailed methods to detect antimicrobial resistance genes and the rationale for selecting isolates for long-read sequencing to investigate plasmid sharing between patients are shown in appendix 1 (p 5). Briefly, *E coli* genomes from all participants were screened for acquired genes encoding antibiotic resistance using Antibiotic Resistance Identification By Assembly (ARIBA; version 2.13.3).^15^ Chromosomal mechanisms of fluoroquinolone resistance were identified by screening isolates for the presence of associated amino acid changes in the quinolone resistance-determining regions of *gyrA* and *parC* alleles.^16,17^ To investigate whether plasmids encoding ESBL genes were shared between patients during the study, one *bla*_CTX-M-15_-positive and *bla*_CTX-M-14_-positive isolate from each sequence type was selected for long-read sequencing using the PacBio Sequel instrument (appendix 1 p 5). In-silico PCR was used for plasmid incompatibility group (replicon) typing.^18^ Geneious (version 11.1) was used for manual annotation and visualisation of complete plasmid sequences. ISFinder and the Basic Local Alignment Search Tool (BLAST; version 2.9.0) were used to identify insertion sequences and transposon fragments. BLAST comparisons visualised in the Artemis Comparison Tool (version 17.0.1) were used for plasmid comparisons (appendix 1 p 5).

### Statistical analysis

The number of positive and negative cultures was assessed in patients who received antimicrobials in the previous 30 days, versus those who did not, with a two-tailed Fisher’s exact test using R (version 3.6.3). Using R, a Mann Whitney *U* test was done to assess the difference in the number of sequenced colonies per stool sample between those with one sequence type and those with multiple sequence types. Plots were created using ggplot2 (version 3.3.1). To further validate the SNP threshold, we used a statistical approach that compared a range of cutoff values (appendix 1 pp 5–6).

### Role of the funding source

The funder of the study had no role in study design, data collection, data analysis, data interpretation, or writing of the report.

## Results

**Figure 1:**
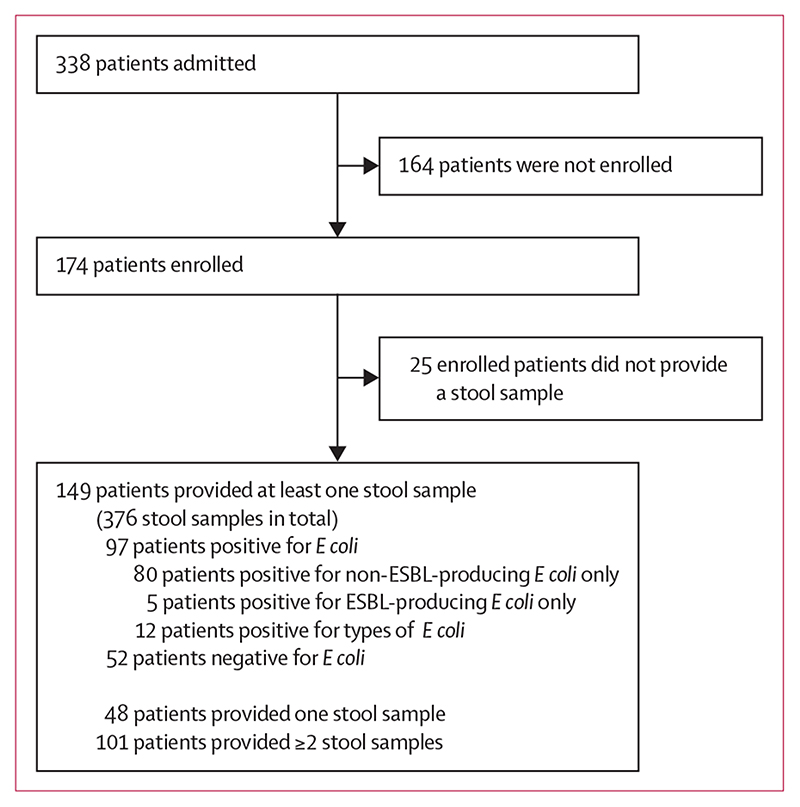
Description of study participants and *Escherichia coli* culture ESBL=extended spectrum β-lactamase.

We recruited 174 (51%) of 338 adult patients admitted to the haematology ward betweeen May 13 and Nov 13, 2015. Of the 174 participants, 92 (53%) were male and 82 (47%) were female, with a median age of 61 years (IQR 49–69; range 19–94 years). Patient characteristics are shown in appendix 1 (p 9).^11^ Most patients (149 [86%]) were able to provide at least one stool sample. 101 patients provided two or more samples. A total of 376 stool samples were collected with a median of three samples (IQR 2–5) per patient. This subset of 149 patients formed the basis for all further analyses. Patients had a median age of 61 years (49–69), with 281 admissions in total (a median of one admission [1–2]), and stayed a median of 16 days (7–27) in hospital, as described previously.^10^ 97 (65%) patients had at least one stool positive for *E coli*, with a total of 152 positive stool samples identified. Most patients (80 [82%] of 97) with positive samples carried only non-ESBL-producing *E coli*, five (5%) carried only ESBL-producing *E coli*, and 12 (12%) carried both (figure 1).

114 (77%) patients received antimicrobials in the 30 days before or during enrolment, or both, including 47 (90%) of 52 patients with a negative *E coli* stool culture and 67 (69%) of 97 patients with a positive culture received antimicrobials in the previous 30 days or at enrolment, or both (p=0·00036; appendix 1 p 6).

To assess *E coli* diversity and putative acquisition, we picked a median of five *E coli* colonies (IQR 5–5; range 1–15; hereafter termed isolates) from each of the 152 primary stool culture plates from 97 patients positive for *E coli*. Overall, whole-genome sequencing was done on 970 isolates (686 non-ESBL-producing and 284 ESBL-producing *E coli)*. From these isolates we identified 90 different sequence types (appendix 1 pp 9, 13; appendix 2 p 1). The most frequently identified sequence types were ST131 (n=14 patients), ST10 (n=9), and ST69 (n=8; appendix 1 p 13), and accounted for 232 (24%) of 970 isolates. 17 (11%) of 149 patients had stool samples positive for ESBL-producing *E coli*, with variation in the presence of genes encoding ESBLs between different sequence types (appendix 1 p 9; appendix 2 p 1). Only *bla*_CIX-M-14_ and *bla*_CTX-M-15_ were present in two or more patients. *E coli* encoding *bla*_CTX-M-15_ was isolated from stools of 12 patients and *bla*_CTX-M44_ from stools of two patients.

**Figure 2:**
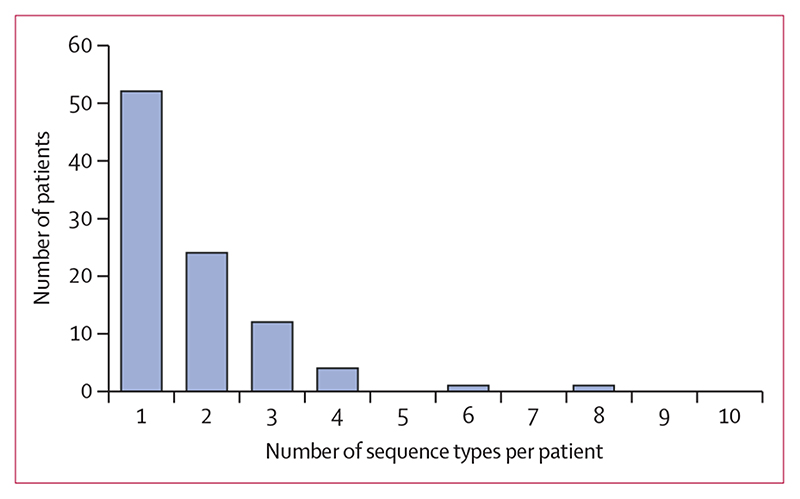
Number of *Escherichia coli* sequence types observed per patient (n=92)

To quantify the amount of within-host *E coli* diversity, we determined the number of different *E coli* sequence types identified from each patient using data on 149 stool samples from 94 patients (excluding three stools from patients for whom only a single *E coli* colony was isolated). Around half (52 [55%]) of 94 patients were positive for a single sequence type. 24 (26%) patients carried two sequence types, 12 (13%) carried three, and six (6%) patients carried four or more sequence types, with a maximum of eight sequence types found in a single patient (figure 2). In a per stool analysis, 104 (70%) of 149 stools contained a single sequence type, 35 (23%) contained two sequence types, and ten (7%) contained more than two sequence types, with a maximum of five sequence types recovered from a single stool. Of the 149 stool samples with multiple isolates sequenced, 104 (70%) contained isolates of the same sequence type, and 45 samples (30%) contained more than one sequence type. There was no significant difference in the number of colonies picked from samples containing a single sequence type (median 5 colonies [IQR 5–5]) and those with multiple sequence types (median 5 [5–14]; 95% CI of the difference between the medians –2·82 × 10^–5^ to –7·15 × 10^−7^; p=0·098).

We then identified sequence types that were isolated from stools obtained from two or more patients, which revealed that 27 sequence types were carried by at least two patients. We questioned whether this finding represented coincidental carriage of the same sequence type or transmission from one patient to another. Acquisition analysis was possible for 71 (70%) of 101 patients who provided at least two stool samples during the study and had at least one positive sample for *E coli*. 30 (42%) patients had putative acquisition of one or more *E coli* sequence types through 50 acquisition events (appendix 1 p 9; appendix 2 p 2). Of the 17 patients positive for ESBL-producing-*E coli*, 13 (76%) were positive for ESBL-producing-*E coli* on their first stool sample, whereas four (24%) had tested positive on follow-up sampling indicating that putative acquisition of ESBL-producing-*E coli* occurred during hospital admission.

**Figure 3:**
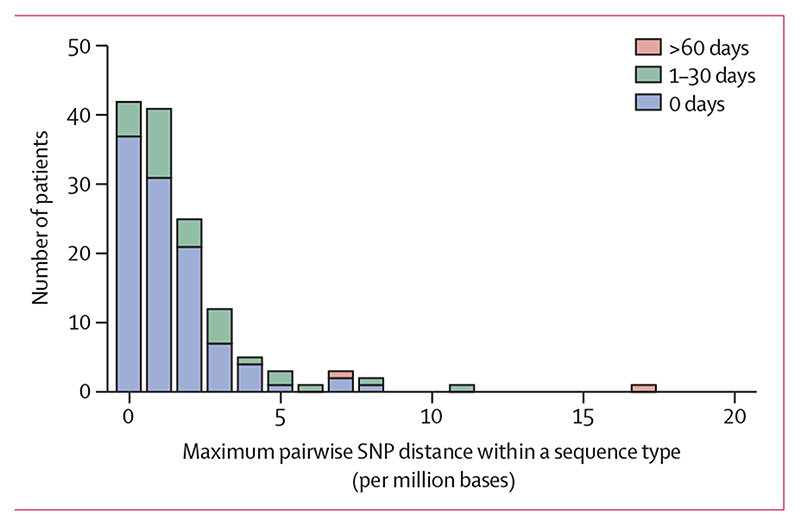
Histogram of maximum pairwise SNP distance (n=92) SNP differences within sequence types from the same patient when at least two isolates of the same sequence type were identified. There were no data points at 31–60 days. SNP=single nucleotide polymorphism.

We used sequence data to define a cutoff of genetic similarity between two genomes that was consistent with *E coli* transmission in the population studied. A core genome pairwise comparison of isolates from the same patient and same sequence type showed a maximum diversity of 17 SNPs (6·8 SNPs per million bases; figure 3), with the exception of three patients who carried isolates which belonged to distinct clades of the same sequence type (different by >300 SNPs, appendix 1 p 9; appendix 2 p 3). The Poisson distribution indicated an upper limit of 25 SNPs (appendix 1 p 14). Having defined two putative but different cutoffs of 17 and 25 SNPs, we used epidemiological information to select the final proposed cutoff. We found that patient pairs with a strong epidemiological link (same bay, room, or ward at the same time or within 7 days) carried isolates that were up to 17 SNPs different, whereas patient pairs carrying isolates 17 to 25 SNPs apart did not have strong epidemiological links. Thus, we selected a 17 SNP cutoff, appreciating that this cutoff is probably more specific but less sensitive than 25 SNPs.

We then applied the 17 SNP cutoff to all 970 *E coli* isolates, reflecting a strictly genomic investigation of putative transmission. We identified ten clusters (defined as containing two or more cases) in 20 patients, four of whom were placed in multiple clusters (table 1; appendix 1 p 15). Strong epidemiological links were found between patients in seven clusters (appendix 1 p 15). The two largest clusters contained seven patients associated with sequence type ST7095 and four patients associated with sequence type ST635 (phylogenetic trees and timelines shown in appendix 1 pp 16–19). These sequence types seemed to have been acquired after admission to hospital (six patients for ST7095 and two for ST635), further supporting hospital acquisition. The remaining eight clusters contained two patients each and were associated with eight different sequence types (ST69, ST131, ST443, ST648, ST1193, ST1196, ST6151, and ST7094).

A serious consequence of *E coli* carriage is the development of a bloodstream infection, which occurred in nine (5%) of 174 patients (281 admissions; appendix 1 pp 10–11) during the study period, equating to around 32 invasive infections per 1000 admissions. All nine patients had a bloodstream infection associated with health-care contact (four hospital acquired and five health-care associated). Seven patients were infected by non-ESBL-producing *E* coli and two by ESBL-producing *E coli*. Seven patients had at least one positive stool cultured and the other two did not provide a stool sample. Four patients provided a stool sample before infection onset. We sequenced 100 colonies from 12 stools from the seven patients (median 15 colonies per patient; range 5–30). The same sequence type was identified in blood and stool samples in four patients (two with ST131, one with ST95, and one with ST1193). Pairwise core genome comparison of these stool and disease-associated *E coli* genomes showed that the blood and stool isolates were very highly related (difference of 0 SNPs).

Over a longer timeframe (from May 13, 2014, to May 13, 2016), we identified 36 additional positive blood cultures from the same two study wards (from 25 patients) with at least one *E coli* isolate available for sequencing. The *E coli* isolates belonged to 18 sequence types, with nine (25%) isolates being ST131 and 12 (33%) ESBL-producing *E coli*. Details of all *E coli* isolates sequenced from bloodstream infections are shown in appendix 2 (p 1).

34 (23%) of 149 patients had *E coli* resistant to ciprofloxacin in stool isolates and the mechanisms of resistance were identified (appendix 1 pp 11; appendix 2 p 4). The types of ESBL-encoding genes we identified and sequence types carrying each type are shown in appendix 1 (pp 7, 9) and appendix 2 (p 1). We selected 31 ESBL-producing *E coli* isolates (21 stool and ten blood cultures) for long-read sequencing. In half of *E coli bla*_CTX-M-15_ cases (eight of 16), the gene was integrated into the chromosome rather than carried on a plasmid, with one further patient carrying an isolate with the gene on both the chromosome and a plasmid. Chromosomal insertion of *bla*_CTX-M-15_ occurred across four STs (ST131, ST443, ST648, and ST90; appendix 1 p 7). We identified two scenarios. The first was that plasmids carrying *bla*_CTX-M-15_ shared few segments (mostly over regions carrying antibiotic resistance genes) of high identity. We also observed the situation where isolates from two patients carried identical plasmids but these isolates differed by 25 or more SNPs and the patients carrying these had weak epidemiological links (appendix 1 p 8).

*bla*_CTX-M-14_ was plasmid-borne (all IncB/O/K/Z) in all five *bla*_CTX-M-14_ positive isolates (four sequence types) from two patients (C062 and C047; table 2). *bla*_CTX-M-14_ positive plasmids from patient C062 were identical (>99% identity over >99% coverage), including plasmids from two different sequence types, consistent with within-host plasmid sharing between sequence types. However, the *bla*_CTX-M-14_ plasmids from C047 showed great diversity and were different to those found in C062. Representative *bla*_CTX-M-14_ carrying plasmids and plasmid comparisons are shown in appendix 1 (pp 11, 20–23) and appendix 2 (p 4).

## Discussion

In this study we extensively examined within-host diversity by serial sampling 94 patients, which showed that almost half of all patients carried more than one sequence type and more than 70% of patients positive for ESBL-producing *E coli* were also positive for non-ESBL-producing *E coli*. This finding indicates that surveillance and outbreak investigations based on single colonies or focused solely on ESBL-producing isolates^19,20^ are likely to underestimate transmission events and the diversity of antimicrobial susceptibility profiles present in a sample. A previous study of 127 genomes from eight children, seven of whom were positive for ESBL-producing *E coli*, identified a median of four sequence types per child (range 1–10).^7^ Analysing seven ESBL-producing *E coli* genomes from three stool samples from one patient with cystic fibrosis identified up to three sequence types per sample.^9^

Diversity was also identified within specific sequence types. A maximum of 17 SNPs were detected per sequence type in each patient, similar to that previously reported (12 SNPs) for ST131 isolated from long-term care facility residents.^8^ To date, few studies have investigated within-host diversity of *E coli* using sequencing, and these studies were small and limited by the inclusion of only ESBL-producing strains.

On the basis of genomic data, we identified that almost a third of patients appeared to acquire one or more *E coli* sequence types through a total of 50 acquisition events. Three of the 50 acquisition events were due to ESBL-producing *E coli*, and in total 34 unique sequence types were acquired. A major strength of our study was the development of a SNP cutoff to support *E coli* transmission in the studied population. Using a cutoff of 17 SNPs we found evidence for transmission that was restricted to small patient clusters. Additionally, we highlight the importance of investigating transmission of non-ESBL-producing *E coli* because eight of ten transmission clusters identified in this study were non-ESBL-producing *E coli*, including the two largest clusters.

The number of *E coli* bloodstream infections are continuously increasing annually but resistance to third-generation cephalosporins accounts for only around 14% of such infections in the UK, which was why we included both ESBL-producing and non-ESBL-producing *E coli*.^21^ By examining all blood cultures positive for *E coli* from the two haematology wards, we identified a diverse collection of invasive strains (19 sequence types) that were predominately non-ESBL producers. These results are consistent with those observed in a national survey of bloodstream infections done between 2001 and 2012 in England, in which less than 15% of invasive isolates were non-susceptible to third-generation cephalosporins annually compared with 17% of ESBL-producing isolates identified in this study.^22^ Our results are in concordance with previous publications that reported ST131 as one of the most frequently recovered lineages from bloodstream infections in the UK.^22–24^ All patients with a bloodstream infection during this study had a genetically distinct strain compared with isolates from other patients recruited to the study, but four of seven patients had highly similar strains in their blood and stool samples, suggesting an endogenous source for the infection.

We also revealed the complexity of investigating the transmission of ESBL-producing genes (*bla*_CTX-M-15_ and *bla*_CTX-M-14_). Previous studies^25^ have shown that characterisation of large plasmids (>50 kilobase pairs) from short-read genome sequence data is challenging due to the presence of repeated sequences. In this study, all ESBL-producing plasmids were fully characterised using long-read sequencing, which provided confidence in our conclusions on plasmid structure, genetic context of ESBL-encoding genes, and transmission. We found that *bla*_CTX-M-15_ was commonly integrated into the chromosome, unlike in previous studies which showed *bla*_CTX-M-15_ to be plasmid-encoded.^26^ Our data show that antimicrobial susceptibility data and plasmid replicon typing is not sufficient to identify plasmid transmission, and long-read sequencing is required to fully understand the dissemination of antimicrobial resistance genes.

Our study has several limitations. We sampled less than 50% of patients admitted to the two haematology wards, and we did not sample the environment or health-care workers. This sampling strategy might lead to underestimated epidemiological links and could explain the absence of links between patients carrying highly related isolates, the absence of genetic links in putative acquisition events, and the inability to identify the source of three putative exogenous infections. Additionally, we did not sequence the full diversity of *E coli* in stool samples, which can lead to some sequence types being misclassified as acquired but instead they might have been present at low abundance in previous samples. We observed that stool samples contained multiple sequence types, but we cannot exclude that these did not contain additional sequence types. Future studies could sequence directly from plate sweeps to capture greater diversity within individuals. We established a SNP cutoff to infer *E coli* transmission in this cohort of patients admitted to hospital. A limitation of this approach is that the directionality of transmission cannot be inferred. Also, combining epidemiological with genomic data to confirm transmission is essential, but this cutoff restricts the number of patients requiring detailed epidemiological follow-up. Additionally, the dataset and method described in this study are of great value to establish a SNP threshold, but more datasets from other settings would be needed to conclude a universal SNP cutoff.

In conclusion, the findings from our study have important implications for carriage, acquisition, and transmission analyses of *E coli*. Our study highlights polyclonal *E coli* colonisation, the value of characterising multiple isolates per sample, and the clinical relevance of *E coli* transmission in the hospital setting. Using the diversity of the same strains from the same host from multipick data we defined a cutoff of clonality that led to the identification of putative nosocomial transmission of *E coli* strains driving carriage and bloodstream infections in patients admitted to hospital. Using long-read sequencing, we identified diverse mechanisms of *bla*_CTX-M-15_ and *bla*_CTX-M-14_ carriage with no evidence of plasmid sharing between patients. High diversity was observed in bacteraemia isolates, but we identified patients with indistinguishable isolates from stool and blood samples, suggesting an endogenous infection. Future interventions to reduce the number of *E coli* bacteraemia should focus on preventing endogenous infections.

## Supplementary Material

Appendix 1

Appendix 2

## Figures and Tables

**Table 1:** Ten patient clusters from genomic analysis of *Escherichia coli* stool isolates

	Sequence type	Acquired sequence type*	SNP distance†
**Cluster 1**			
C011	ST7095	Yes	First case detected
C016	ST7095	Yes	2–6
C095	ST7095	Yes	2–3
C098	ST7095	Yes	0–2
C100	ST7095	Yes	5–7
C104	ST7095	Yes	2–4
D058	ST7095	No	1–3
**Cluster 2**			
D013	ST635	No	First case detected
C100	ST635	Yes	0
D038	ST635	No	3
D045	ST635	Yes	1–2
**Cluster 3**			
C031	ST1193	No	First case detected
C043	ST1193	Yes	0–2
**Cluster 4**			
C023	ST1196	No	First case detected
C035	ST1196	No	0–7
**Cluster 5**			
C022	ST131	No	First case detected
C027	ST131	No	0
**Cluster 6**			
C043	ST6151	No	First case detected
C031	ST6151	Yes	0–2
**Cluster 7**			
C031	ST648	No	First case detected
C043	ST648	Yes	0–1
**Cluster 8**			
C096	ST69	No	First case detected
C100	ST69	Yes	0–1
**Cluster 9**			
C059	ST7094	No	First case detected
D058	ST7094	Yes	0–1
**Cluster 10**			
C005	ST443	No	First case detected
D030	ST443	No	8–11

Patient identification codes are indicated within each cluster. SNP=single nucleotide polymorphism.

* Patients were previously negative for *E coli* or acquired a new sequence type. †SNP distance range refers to the minimum-maximum SNPs between the isolate from that patient and others in the cluster.

**Table 2:** Plasmids encoding *bla*_CTX-M-15_ or *bla*_CTXM-14_ from PacBio sequencing

	Sample identification	Patient identification	Sample type	Sequence type	Plasmid size, bP	Incompatibility group	Phenotypic resistance*	Antimicrobial resistance genes on plasmid
LR595882	3546	B005†	Blood	648	152 153	IcFIA, IncFIB, IncFII	Cefotaxime, ceftazidime, amoxicillin, ciprofloxacin, gentamicin, piperacillin with tazobactam	*bla*_CTX-M-15_; *bla*_TEM-1_; *aac(3)-IIa; dfrA17; sul1; tetB; mphA; aadA5; strAB; ermB*
LR595874	3547	B005†	Blood	648	152 153	IcFIA, IncFIB, IncFII	Cefotaxime, ceftazidime, amoxicillin, ciprofloxacin, gentamicin, piperacillin with tazobactam	*bla*_CTM-15_; *bla*_TEM-1_*aac(3)-IIa; dfrA17; sull; tetB; mphA; aadA5; strAB; ermB*
LR595875	3580	B006†	Blood	131	111 743	IncFIB	Cefotaxime, ceftazidime, amoxicillin, gentamicin	*bla*_CTM-15_
LR595876	3550	C042†	Blood	2006	170 000	IncFIA, IncFIB, IncFII	Cefotaxime, ceftazidime, amoxicillin, ciprofloxacin, gentamicin	*bla*_CTX-M-15_; *bla*_oxA-1_; *aac(3)-IIa; aac6_prime-Ib-cr; dfrA17; sul1; tetB; mphA; aadA5*
LR595878	3271	C025	Stool	1723	111 381	IncFIB	Cefotaxime, ceftazidime, ciprofloxacin	*bla*_CTM-15_
LR595886	2898	C065	Stool	131	164 328	IncFIA, IncFII, IncN	Cefotaxime, ceftazidime, ciprofloxacin	*bla*_CTX-M-15_ *bla*_OXA-1_ *aac6_prime-Ib-cr; dfrA17; sul1; mphA; aadA5; tetA*
LR595884	2981	C071	Stool	131	61 991	IncFIA, IncFIB	Cefotaxime, ceftazidime, amikacin, amoxicillin, ciprofloxacin, gentamicin	*bla*_CTX-M-15_; *bla*_oxA-1_; *aac(3)-IIa; aac6_prime-Ib-cr; dfrA17; suì1; mphA; aadA5; tetA*(x2); *strAB*
LR595879	3060	C071	Stool	131	69 882	IncFIA, IncFIB	Cefotaxime, ceftazidime, amikacin, amoxicillin, ciprofloxacin, gentamicin, piperacillin with tazobactam	*bla*_CTX-M-15_; *bla*_oxA-1_(x2); *aac(3)-IIa; aac6_prime-Ib-cr*; *dfrA17*; *sul1*; *mphA*; *aadA5*; *tetA*(x3); *strAB*
LR595890	2766	D038	Stool	1723	111 381	IncFIB	Cefotaxime, ceftazidime, ciprofloxacin	*bla*_CTM-15_
LR595881	3125	D050	Stool	7097	81 285	IncFIB	Cefotaxime, ceftazidime	*bla*_CTX-M-15_; *qnrS1; dfrA14; sul2;* bla_TEM-1_
LR595877	2656	C047	Blood	156	111 594	IncB/O/K/Z	Cefotaxime, gentamicin	*bla*_CTX-M-14_; *aac(3)-IIa; dfrA17; sul1*; *mphA*; *aadA5*
LR595889	2604	C047	Blood	428	94 296	IncB/O/K/Z	Cefotaxime	*bla*_CTX-M-14_
LR595871	2656	C047	Blood	428	94 061	IncB/O/K/Z	Cefotaxime	*bla*_CTX-M-14_
LR595888	2887	C062	Stool	3877	96 306	IncB/O/K/Z	Cefotaxime	*bla*_CTX-M-14_
LR595880	2978	C062	Blood	131	96 305	IncB/O/K/Z	Cefotaxime, amoxicillin, ciprofloxacin, gentamicin	*bla*_CTX-M-14_
LR595872	3877	C062	Stool	3877	96 306	IncB/O/K/Z	Cefotaxime, amoxicillin	*bla*_CTX-M-14_

* Antimicrobial non-susceptibility detected by VITEK2.

† Blood samples taken before and after the study.

## Data Availability

The sequence data generated in this study have been submitted to the NCBI BioProject database (https://www.ncbi.nlm.nih.gov/bioproject/) under accession numbers PRJEB19918 and PRJEB21499, and individual accession numbers for Illumina and PacBio data are listed in appendix 1 (pp 9, 12) and appendix 2 (p 1).

## Research in context

### Evidence before this study

We searched PubMed for studies published in English from database inception to March 17, 2020, using the terms *“Escherichia coli”*, “whole genome sequencing”, “transmission”, and “hospital”. We excluded reviews and included articles in which whole-genome sequencing was applied to study *E coli* transmission in human populations in a hospital setting (15 of 75 studies). 12 of 15 studies were focused on carbapenem or colistin resistance and were not evaluated further. Of the three remaining studies, one focused on the national epidemiology of a single clone (ST410) in Denmark and was based on 127 whole-genome sequenced isolates. Five possible regional outbreaks were identified using ten or less single nucleotide polymorphisms (SNPs). In a second study done in Denmark, whole-genome multilocus sequence typing (MLST) was used to distinguish between epidemiologically related and unrelated isolates of extended spectrum β-lactamase (ESBL)-producing *E coli*. Isolates obtained from the same patient, belonging to the same MLST, and cultured within 30 days were defined as epidemiologically related. In a third study, transmission of *E coli* among patients in haematology and oncology departments of German hospitals was investigated using core-genome MLST, in which closely related isolates were defined as having a maximum of ten allele differences.

### Added value of this study

Our findings capture what happens during routine care, beyond the current bacterial genomics literature which largely focuses on outbreak investigations. We show that surveillance and outbreak investigations based on single colonies are likely to underestimate transmission events and the diversity of antimicrobial susceptibility profiles present in a sample. Our study also adds to the existing evidence on suitable methods to determine transmission events. We established a genome-based SNP threshold to infer *E coli* transmission in the study population by comparing SNP distances of isolates from the same host and combining these with epidemiological data. We identified transmission clusters involving predominately patients with non-ESBL *E coli*, which would be missed by other investigations focused on antimicrobial-resistant *E coli*. Using long-read sequencing, we were able to accurately study the transmission of antimicrobial resistance genes conferring resistance to cephalosporin drugs (ESBLs) and plasmids. Although *E coli* from patients carried the same genes conferring resistance, the genes were rarely carried on the same plasmids as those found in other patient samples. This finding would not have been identified using short-read sequencing. By comparing *E coli* isolates from blood and stool samples of individual patients we identified indistinguishable isolates, suggesting endogenous infection.

### Implications of all the available evidence

Our study highlights polyclonal *E coli* colonisation, the pathogenesis of extraintestinal *E coli* infection (endogenous *vs* exogenous), and the clinical relevance of *E coli* transmission in the hospital setting. Our findings challenge the widely accepted notion of plasmid spread, at least for *E coli*, in this setting. Interventions to reduce *E coli* bacteraemia should aim to prevent endogenous infections, which were the main source of infection.
