# Supplementary material for: Defining nosocomial transmission of Escherichia coli and antimicrobial resistance genes: a genomic surveillance study
Source: Lancet Microbe. Author manuscript; Available in PMC 2021 Sep 6. (PMC8410606; doi:10.1016/S2666-5247(21)00117-8)
Supplement: Appendix 1 [file EMS130926-supplement-Appendix_1.pdf]

THE LANCET
Microbe

Supplementary appendix 1

This appendix formed part of the original submission and has been peer reviewed. We post it as supplied by the authors.

Supplement to: Ludden C, Coll F, Gouliouris T, et al. Defining nosocomial transmission of *Escherichia coli* and antimicrobial resistance genes: a genomic surveillance study. *Lancet Microbe* 2021; published online July 5. https://doi.org/10.1016/S2666-5247(21)00117-8.

Supplementary appendix

Title: Defining nosocomial transmission of *Escherichia coli* and antimicrobial resistance genes: a genomic surveillance study

Authors: Catherine Ludden, Francesc Coll, Theodore Gouliouris, Olivier Restif, Beth Blane, Narender Kumar, Grace A. Blackwell, Plamena Naydenova, Charles Crawley, Nicholas M. Brown, Julian Parkhill, Sharon J. Peacock

APPENDIX

1. SUPPLEMENTARY METHODS
2. SUPPLEMENTARY RESULTS
3. SUPPLEMENTARY TABLES
4. SUPPLEMENTARY FIGURES
5. SUPPLEMENTARY REFERENCES

1. SUPPLEMENTARY METHODS

Sample collection, bacterial isolation and antimicrobial susceptibility testing

Stool samples were obtained using a sterile scoop (Thermo Scientific Sterilin™ Polystyrene Universal Containers, Thermo Fisher Scientific, Paisley, UK). Stool samples were refrigerated following collection and processed within 24 h on weekdays or 72 h if collected over the weekend. A pea-sized amount was cultured onto Brilliance UTI Chromagar (Oxoid, Basingstoke, UK) to detect all *E. coli* and Brilliance™ ESBL agar (Oxoid, Basingstoke, UK) to detect extended-spectrum beta-lactamase (ESBL) producing *E. coli*. Enrichment cultures were also performed to detect ESBL-producing *E. coli* by adding approximately 0.2 g of stool to 10 mL of Tryptic Soy Broth (Sigma, Dorset, UK) containing cefpodoxime 1 µg/mL (Oxoid), vortexed, and incubated with shaking at 150 rpm at 37 °C in air overnight. The following day, 200 µL of suspension was inoculated onto Brilliance™ ESBL agar (Oxoid, Basingstoke, UK). Up to 15 *E. coli* colonies were picked from primary cultures of positive stool samples and selected for sequencing (10 putative ESBL *E. coli* colonies and 5 non-ESBL *E. coli* when positive for both) to detect genetic diversity. In the case that there were less than 5 non-ESBL *E. coli* colonies identified, extra ESBL-*E. coli* colonies were selected.

During the 6-month study, blood cultures were obtained from study patients by treating doctors following hospital protocols and processed by the diagnostic laboratory. Blood culture sets consisting of three bottles (aerobic, anaerobic and FAN, BacT/ALERT, bioMérieux) were obtained peripherally and/or centrally and incubated for up to 5 days. Positive growth was sub-cultured onto a range of primary media. In the event that these were positive for E. coli, the primary sub-culture plates were identified and retrieved from the diagnostic laboratory and up to 12 colonies cultured from each blood sample collected over the 6-month study were selected for sequencing. For blood cultures positive for *E. coli* retrieved from patients residing in the haematology wards in the 12 months before (May 2014 – May 2015) and six-months after the study (November 2015-May 2016), one colony was obtained from culture of the freezer archive for sequencing. Antimicrobial susceptibility testing was determined for

all isolates using the N206 card on the Vitek 2 instrument (bioMérieux, Marcy l'Étoile, France) calibrated against EUCAST breakpoints.

Sequencing and bioinformatic analyses

In total, 1106 isolates were selected for sequencing from stool (n=983) and blood (n=123). Bacterial genomic DNA was extracted using the QIAxtractor (Qiagen, Hilden, Germany) according to the manufacturer's instructions. Library preparation was conducted according to the Illumina protocol and sequenced on an Illumina HiSeq2000 (Illumina, San Diego, CA, USA) with 125-cycle paired-end reads. Multiple assemblies were created using VelvetOptimiser v2.2.5 (https://github.com/tseemann/VelvetOptimiser) and Velvet v1.2 [1]. An assembly improvement step was applied to the assembly with the best N50 and contigs were scaffolded using SSPACE [2] and sequence gaps filled using GapFiller[3]. Assemblies were annotated using Prokka v1.5 [4] and a genus specific database from RefSeq[5]. In-silico ST was identified for all sequenced isolates using the MLST sequence archive (https://enterobase.warwick.ac.uk). Thirteen isolates were excluded due to failed library preparation (n=1), poor assembly (n=1), species misidentification (n=9), or belonging to cryptic clades in a maximum-likelihood phylogenetic tree based on single nucleotide polymorphisms (SNPs) in the core genes (n=2). Following QC of the remaining 1093 genomes (n=970 from stool, 123 from blood, Supplementary Table 2), all were then assembled using SPAdes 3.11.0 and the assembly pipeline generated an average total length of 5075255 bp (range 4434702 - 5829996 bp) from 76 contigs (range 16 - 441) with an average contig length of 79524 bp (range 13220 - 294438 bp) and an N50 of 245702 bp (range 49703 - 1188737 bp).

The 1093 *E. coli* genomes were mapped against the *E. coli* LT632320 reference strain using SMALT v0.7.4 (http://www.sanger.ac.uk/science/tools/smalt-0). The *E. coli* core genome was derived using Roary version 1.7.1 [6] using a BLASTP identity of 90% and core definition of 99%. Whole-genome alignments were created by calling nucleotide alleles along the LT632320 reference genome. Only core genes present in the reference genome were retained to calculate pairwise SNP distances in core-genome alignments using pairsnp v0.0.1 (https://github.com/gtonkinhill/pairsnp). In addition, we

investigated whether SNPs associated with recombination would affect transmission analyses by identifying the pairwise distance between isolates from the same ST in the same patient with and without removing regions containing recombination (100bp regions with >= 3 SNPs) (Supplementary Table 3). SNPs (n=270,702,217) were extracted from the core-genome alignment and used to construct a maximum likelihood phylogeny using RAxML version 7.0.3[7] with 100 bootstraps and a midpoint root (Supplementary Figure 1).

One isolate assigned to each of ST7095 and ST635 were selected for long-read sequencing. These isolates were selected as they had the assembly with the best N50 and were located in the largest clade of each ST in the core-genome phylogeny. DNA was extracted for the two representatives using the Qiagen MagAttract HMW DNA kit (Qiagen, Hilden, Germany) and sequenced using the PacBio Sequel instrument. De-multiplexed sequences reads were assembled using HGAP v4 with SMRT® Link v5.1.0 (https://www.pacb.com/documentation/analysis-procedure-multiplexed-microbial-assembly-with-smrt-link-v510/) and circularized using Circlator v1.5.3[8]. Isolate genomes belonging to these two STs were mapped against the chromosome of the corresponding PacBio reference (Accession number LR536431and accession number LR536430, respectively) using SMALT v0.7.4 (http://www.sanger.ac.uk/science/tools/smalt-0). As before, SNPs were identified in the core-genome alignment, recombination events removed using Gubbins v1.4.10[9] and a maximum-likelihood phylogeny created using RAxML version 7.0.3[7] with 100 bootstraps and a midpoint root.

Determining SNP cut-off to detect recent transmission based on within-host diversity

The genomes of multiple *E. coli* isolated from the same patient were used to ascertain *E. coli* within-host diversity, as this is the diversity that could potentially be transmitted between hosts. The number of SNPs accumulated by *E. coli* STs in the same patient during the study period (6 months) was quantified to inform transmission analyses. This was based on analysis of STs with at least two isolates from the same patient (total of 139 patient-ST combinations from 94 patients). Isolates belonging to the same ST in the same patient differed by no more than 17 SNPs (Figure 3), after discarding three outliers. However, all pairs within 12-17 SNPs came from a small number of patients who had been sampled

repeatedly more than a month apart. To validate the SNP threshold using a statistical approach we modelled the distribution of within-patient SNPs as a mixture of Poisson distributions. Based on Akaike's Information Criterion, the best model was a zero-inflated mixture of two Poisson distributions. We then computed the probability of drawing the observed pairs of between-patient isolates, which fell sharply below 1% for pairs distant by more than 25 SNPs (Supplementary Figure 2). This allowed us to exclude pairs of patients distant by more than 25 SNPs. We concluded that 17 SNPs was a suitable cut-off to detect transmission of *E. coli* STs between patients in this study.

Detection of antimicrobial resistance and mobile elements

To investigate if plasmids encoding ESBL genes were shared between patients during the study, one $bla_{CTX\text{-}M\text{-}15}$ and $bla_{CTX\text{-}M\text{-}14}$ isolate from each ST per positive sample (n=16 and n=6, respectively) were selected. One isolate from each ESBL (all $bla_{CTX\text{-}M\text{-}15}$) positive blood culture (n=9) from 6 patients obtained from the study wards 1 year prior to the study and 6 months after the study were selected for long-read sequencing using the PacBio Sequel instrument. Analysis was limited to these as they were the only ESBL genes found in samples from more than 1 patient. Chromosomal mechanisms of fluoroquinolone resistance were identified by screening all ciprofloxacin resistant isolates for mutations affecting serine 83 (S83) and aspartate 87 (D87) in *gyrA*, and serine 80 (S80) and glutamate 84 (84) in *parC*, as previously described[10-12] (Supplementary Table 6). Acquired genes encoding antibiotic resistance were identified using Antibiotic Resistance Identification By Assembly (ARIBA)[13] with default length and match thresholds of 95% and 90% respectively, using ResFinder database (https://cge.cbs.dtu.dk/services/ResFinder/). Genes reported as fragmented, partial or interrupted were excluded.

Statistical analysis

We fitted a mixture of Poisson distributions to the within-patient SNP data. We computed the maximum likelihood of models with a range of 1 to 4 Poisson and zero-inflated Poisson mixtures and selected the most suitable model using Akaike Information Criterion (AIC). Next, we computed the likelihood of the selected model for the between-patient SNP dataset across a range of cut-off values between 13-30

SNPs. See Supplementary Figure 2 for a complete description and visualisation of the analysis. For each candidate cut-off value, we compared the maximum likelihood value for the between-patient data with the distribution of likelihood values obtained by sampling 10,000 random datasets of the same size from the mixture model. If the likelihood value from the data fell outside of the 99th percentile of the randomised likelihood distribution from the model, we deemed the between-patient SNP distribution to be statistically distinct from the within-patient data. The optimal cut-off value was determined as the smallest cut-off for which the two distributions became statistically distinct.

2. SUPPLEMENTARY RESULTS

Isolation of E. coli and antimicrobial administration

Empiric treatment for febrile neutropenic patients at the time of the study was meropenem. Prophylactic antibiotics were prescribed for high-risk neutropenic patients (ciprofloxacin) and haemopoietic stem cell transplantation (HSCT) recipients (co-trimoxazole). 114/149 participants (77%) received antimicrobials in the previous 30 days and/or on enrolment. A comparison of the rate of *E. coli* stool culture positivity showed that 47/52 patients (90%) with negative culture(s) and 67/97 patients (69%) with a positive culture(s) received antimicrobials in the previous 30 days and/or on enrolment (p=0.00036).

Implications of E. coli carriage and transmission

To add context to the genetic diversity of *E. coli* causing bloodstream infections over the 6-month study, we identified all patients with *E. coli* bloodstream infection who were admitted to the two study wards over a longer timeframe of two years (May 2014 to May 2016, encompassing the 6-month study). This identified 36 additional positive blood cultures from 25 patients, with all 36 cultures having at least one *E. coli* isolate available for sequencing. Sequencing confirmed a diverse *E. coli* population with 18 different STs identified, although 9/36 (25%) infections were caused by ST131. A third of infections

(12/36, 33%) were caused by ESBL-producing *E. coli*, of which 8 (67%) were ST131, highlighting the role of ST131 in multidrug-resistant invasive infections.

Analysis of putatively transmissible antimicrobial resistance determinants

34/149 (23%) patients had *E. coli* resistant to ciprofloxacin in stool isolates (Supplementary Table 6). Further analysis of resistance genes focused on ESBL (extended spectrum beta-lactamases). Two hundred and eighty-four ESBL-positive stool isolates from 17/149 patients (11.4%) had phenotypic resistance to third generation cephalosporins (12 of whom also carried non-ESBL *E. coli*). The ESBL genes were diverse in type ($bla_{CTX-M-15}$=194, $bla_{CTX-M-14}$=51, $bla_{CTX-M-27}$=15, bla_{SHV-12}=14, $bla_{CTX-M-1}$=10). Only $bla_{CTX-M-14}$ and $bla_{CTX-M-15}$ were present in two or more patients. *E. coli* encoding $bla_{CTX-M-15}$ was isolated from stools from 12 patients and was carried by 6 different STs (Supplementary Table 2). *E. coli* encoding $bla_{CTX-M-14}$ was isolated from two patients and was carried by 4 different STs (Supplementary Table 2).

We used long-read sequencing to further investigate plasmids and the genetic context of ESBL genes. We sequenced one representative isolate from each patient, and from each ST positive for $bla_{CTX-M-15}$ or $bla_{CTX-M-14}$ (including blood and stool). This totalled 31 *E. coli* isolates (21 stools and 10 blood cultures) from 18 patients, including 25 $bla_{CTX-M-15}$ and 6 $bla_{CTX-M-14}$ isolates from 16 and 2 patients, respectively. In half of *E. coli* $bla_{CTX-M-15}$ cases (8/16), the gene was integrated into the chromosome rather than carried on a plasmid, with one further patient carrying an isolate with the gene on both the chromosome and a plasmid (B006). Chromosomal insertion of $bla_{CTX-M-15}$ occurred across four STs (ST131, ST443, ST648 and ST90). Three patients carried a mixture of $bla_{CTX-M-15}$ positive and negative isolates (1 pair =0 SNPs different, 1 pair =2 SNPs, 1 pair=327 SNPs) belonging to the same ST. In the remaining five patients, $bla_{CTX-M-15}$ was carried on nine different plasmids (See exemplars in Supplementary Figure 6). The plasmids carrying the $bla_{CTX-M15}$ gene were diverse both in the replicons and the antibiotic resistance genes they contained, as well as their size (Table 2).

Plasmids LR595884 and LR595879 from the same patient (C071) shared identical sequence, except that LR595879 contained an additional 7891 bp segment that is not found in LR595884 (Supplementary Figure 6a). Plasmid LR595881 from a second patient (D050), and LR595886 from patient C065 shared segments of high identity with LR595879, though mostly over regions carrying antibiotic resistance genes, indicating shared mobile genetic elements rather than sharing whole plasmids (Supplementary Figure 6abd).

In the two remaining patients with $bla_{CTX\text{-}M15}$ *E. coli* isolated from stool (D038 and C025, see Table 2), patients carried the same ST (ST1723) and an identical 111 kb bacteriophage-like plasmid (IncFIB type). The replicon of this plasmid was identical to a previously reported bacteriophage-like plasmid (accession no. HG530657)[14]. This is unlikely to be a direct bacterial transmission, as the ST1723 isolates themselves were 25 SNPs different and had only weak strong epidemiological link, i.e. were residing in the study hospital within the same 7 days, but were located on different wards. Similarly, all plasmids encoding $bla_{\text{CTX-M-15}}$ found in blood samples before and after the 6-month study (Table 2 and Supplementary Figure 6a-d) were different to plasmids identified during the 6-month study. Thus, we found no evidence for horizontal spread of plasmids carrying $bla_{\text{CTX-M-15}}$ in our patient cohort.

3. SUPPLEMENTARY TABLES

Supplementary Table 1. Characteristics of study participants at time of enrolment

Variable	All participants (n=174)	Participants with sampled stool (n=149)	Participants with no sampled stool (n=25)	P value
Age (years), median (IQR)	61 (49-69)	61 (49-68)	58 (45-70)	0.90
Male	92 (53)	80 (54)	12 (48)	0.60
Transfer from another hospital	33 (19)	28 (19)	5 (20)	0.89
Hospitalization previous year<br>CUH<br>Other hospital	112 (64)<br>75 (43)<br>70 (40)	94 (63)<br>64 (43)<br>58 (39)	18 (72)<br>11 (44)<br>12 (48)	0.39
Number of admissions to CUH previous year, median (IQR)	0 (0-2)	0 (0-2)	0 (0-2)	0.60
Duration of hospitalization previous year (days), median (IQR)	0 (0-20)	0 (0-22)	0 (0-7)	0.78
Haematological malignancy[a]	160 (92)	141 (95)	19 (76)	<0.01
Acute myeloid leukaemia	45 (26)	39 (26)	6 (24)	
Acute lymphoblastic leukaemia	10 (6)	9 (6)	1 (4)	
Non-Hodgkin's lymphoma	57 (33)	50 (34)	7 (28)	
Multiple myeloma	32 (18)	29 (19)	3 (12)	
Myelodysplastic syndrome	12 (7)	11 (7)	1 (4)	
Hodgkin's disease	5 (3)	4 (3)	1 (4)	
Other	2 (1)	1 (0.6)	1 (4)	
New haematological diagnosis	45 (26)	40 (27)	5 (20)	0.63
Haematopoietic stem cell transplant prior to enrolment	26 (15)	25 (17)	1 (4)	0.13
Allogeneic	16 (62)	15 (60)	1 (100)	
Autologous	10 (38)	10 (40)	0 (0)	
Haematopoietic stem cell transplant during study period	54 (31)	51 (34)	3 (12)	0.03
Allogeneic	29 (54)	26 (51)	3 (100)	
Autologous	25 (46)	25 (49)	0 (0)	

Data are presented as number (%) of patients unless indicated otherwise. [a] Three patients had two haematological conditions. Abbreviations: CUH, Cambridge University Hospitals.

Supplementary Table 2. Details of all *E. coli* isolates sequenced from stool carriage and bloodstream infections (see excel S2).

Supplementary Table 3. Epidemiological and genetic links associated with strain acquisition during the study (see excel S3).

Supplementary Table 4. Pairwise comparisons of SNPs in the core genome for isolates from the same ST per patient before and after removing recombination (see excel S4)

Supplementary Table 5. Characteristics of patients with *E. coli* bloodstream infection during the 6-month study

Patient	D019	D038	D053	D066	C018[a]	C062[a]	C074[a]	D015[b]	D032[b]
**Gender**	Female	Male	Male	Female	Male	Male	Male	Female	Female
**Age**	73	73	44	86	73	57	57	49	49
**Onset**	HCA	HCA	HA	HA	HCA	HA	HA	HCA	HCA
**Duration of admission (days)**	8	Treated as outpatient	44	42	9	20	19	1	4
**Haematological malignancy**	MDS	AML	NHL	None	NHL	MM	MM	None	NHL
**Stem cell transplant (type)**	No	No	No	No	No	Autograft	Autograft	No	No
**Neutropenia at time of infection**	Yes	Yes	Yes	No	No	Yes	Yes	No	No
**Source of infection**	Neutropenia	Mucositis/ neutropenia	UTI	UTI	UTI	Mucositis/ neutropenia	CVC	CVC	UTI
**Day of positive culture post admission**	0	Treated as outpatient	25	5	0	12	10	Taken 1 day prior to admission	0
**ESBL status**	Positive	Negative	Negative	Negative	Negative	Positive	Negative	Negative	Negative
**Ciprofloxacin susceptible/Cip resistant**	Cip resistant	Cip susceptible	Cip resistant	Cip susceptible	Cip susceptible	Cip resistant	Cip resistant	Cip susceptible	Cip susceptible
**Prophylaxis recorded at time of bacteraemia.**	Ciprofloxacin	No	Co-trimoxazole	No	No	Ciprofloxacin	Ciprofloxacin	No	No

**Survived at discharge**	Yes	Yes	Yes	Yes	Yes	Yes	Yes	Yes	Yes
**Stool taken**	Yes	Yes	Yes	Yes	Yes	Yes	Yes	No	No
**ST in blood (SNP distance for matching infection/stool)**	131 (0)	95 (0)	1193 (0)	131(0)	69[a] in blood (N/A)	131[a] in blood (N/A)	648[a] in blood (N/A)	4959[b] in blood (N/A)	73[b] in blood (N/A)

a Study patients with different ST in blood and stool; b Study patients with no stool sample received; NHL = Non-Hodgkin's lymphoma; MM = multiple myeloma; MDS = myelodysplasia; AML - Acute myeloid leukaemia; ALL – Acute lymphoblastic leukaemia; NHL - Non-Hodgkin's lymphoma; UTI – Urinary tract infection; CVC – Central venous catheter; HA – Hospital acquired; HCA – Healthcare associated

Supplementary Table 6. Mechanisms of ciprofloxacin resistance in blood and stools samples (see excel S6).

Supplementary Table 7. Comparison of plasmids encoding $bla_{CTX-M-14}$ from all positive samples (n=6)

	C047<br>LR595871 (94,061)	C047<br>LR595889<br>(94,296)	C047<br>LR595877<br>(111,594)	C062<br>LR595880<br>(96,305)	C062<br>LR595888<br>(96,306)	C062<br>LR595872<br>(96,306)
C047<br>LR595871<br>(94,061)	-	93461 bp<br>953 SNPs<br>99% cov, 98.62% ID	96462 bp<br>729 SNPs<br>99% cov, 99.33% ID	87008 bp<br>1166 SNPs<br>95% cov, 98.34% ID	87779 bp<br>1164 SNPs<br>95% cov, 98.34% ID	87738 bp<br>1166 SNPs<br>95% cov, 98.34%ID
C047<br>LR595889<br>(94,296)	75907 bp<br>473 SNPs<br>99% cov, 98.62% ID	-	95631 bp<br>574 SNPs<br>100% cov, 98.96% ID	86881 bp<br>1031 SNPs<br>95% cov, 98.40% ID	86245 bp<br>1025 SNPs<br>95% cov, 98.40% ID	86275 bp<br>1024 SNPs<br>95% cov, 98.40% ID
C047<br>LR595877<br>(111,594)	73313 bp<br>230 SNPs<br>86% cov, 99.33% ID	79075 bp<br>567 SNPs<br>86% cov, 98.96% ID	-	87938 bp<br>535 SNPs<br>80% cov, 99.40% ID	87248 bp<br>544 SNPs<br>80% cov, 99.39% ID	87399 bp<br>542 SNPs<br>80% cov, 99.40% ID
C062<br>LR595880<br>(96,305)	69706 bp<br>651 SNPs<br>92% cov, 98.34 ID	87017 bp<br>991 SNPs<br>93% cov, 98.4% ID	87283 bp<br>492 SNPs<br>93% cov, 99.4% ID	-	96358 bp<br>7 SNPs<br>100% cov, 100% ID*	95205 bp<br>12 SNPs<br>100% cov, 100% ID*
C062<br>LR595888<br>(96,306)	69731 bp<br>653 SNPa<br>92% cov, 98.34 ID	86330 bp<br>991 SNPs<br>93% cov, 98.4% ID	87336 bp<br>504 SNPs<br>93% cov, 99.39% ID	96358 bp<br>7 SNPs<br>100% cov, 100% ID*	-	94640 bp<br>8 SNPs<br>100% cov, 100% ID*
C062<br>LR595872<br>(96,306)	70466 bp<br>660 SNPs<br>92% cov, 98.34% ID	86406 bp<br>994 SNPs<br>93% cov, 98.4% ID	87530 bp<br>505 SNPs<br>93% cov, 99.4% ID	95925 bp<br>5 SNPs<br>100% cov, 100% ID*	94637 bp<br>7 SNPs<br>100% cov, 100% ID*	-

Cov = coverage, ident = identity, bp= matched base pairs, SNPs = single nucleotide polymorphisms, yellow shading = C047 plasmids, blue shading = C062 plasmids, green shading = plasmids between patients

* Identical sequence though different order of segments of DNA at the shufflon.

4. SUPPLEMENTARY FIGURES

Supplementary Figure 1. *E. coli* diversity in blood and stool

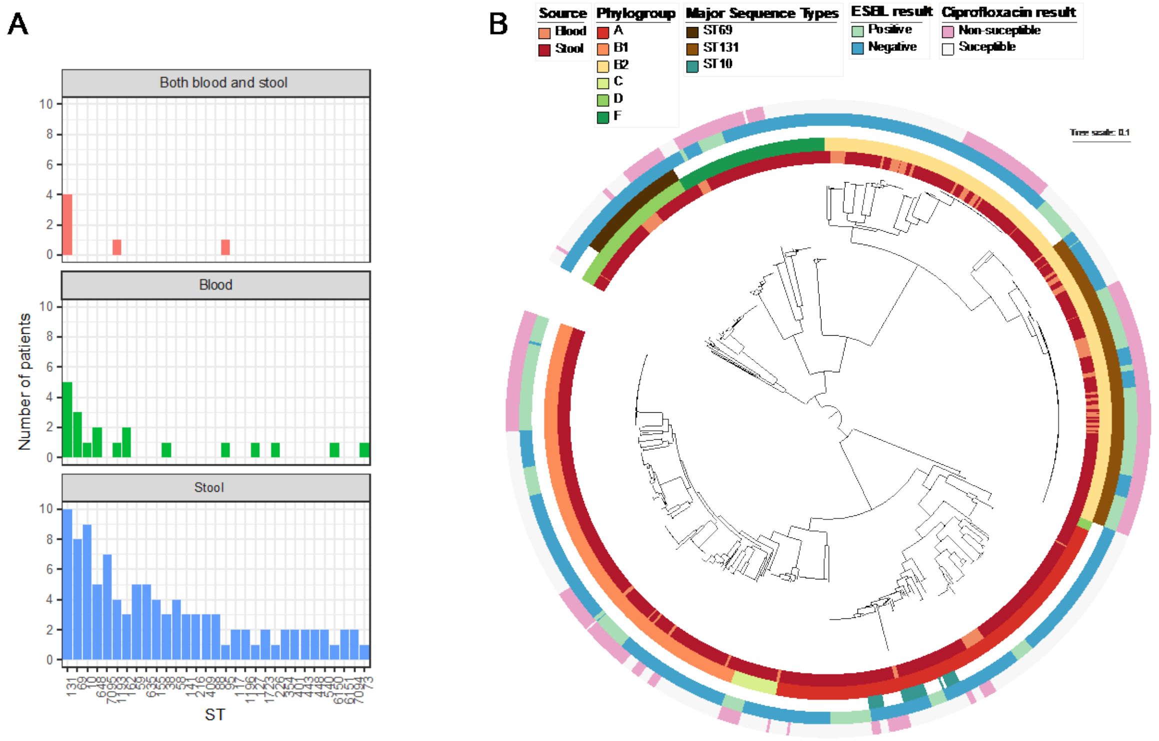

(A) Frequency of multilocus sequence types (STs) identified in *E. coli* isolated from blood and stool. Only those STs found in more than one patient are shown (n=31) **(B)** Maximum likelihood phylogenetic tree based on SNPs in the genes core of 970 *E. coli* isolates cultured from stools (n=970) and blood (n=123) annotated by source, phylogroup, major sequence types, ESBL positive/negative and ciprofloxacin susceptibility. Red circles on branches are bootstrap support values from 100 replicates if equal to or larger than 70%.

Supplementary Figure 2. Defining a genetic threshold to infer *E. coli* transmission based on a statistical approach.

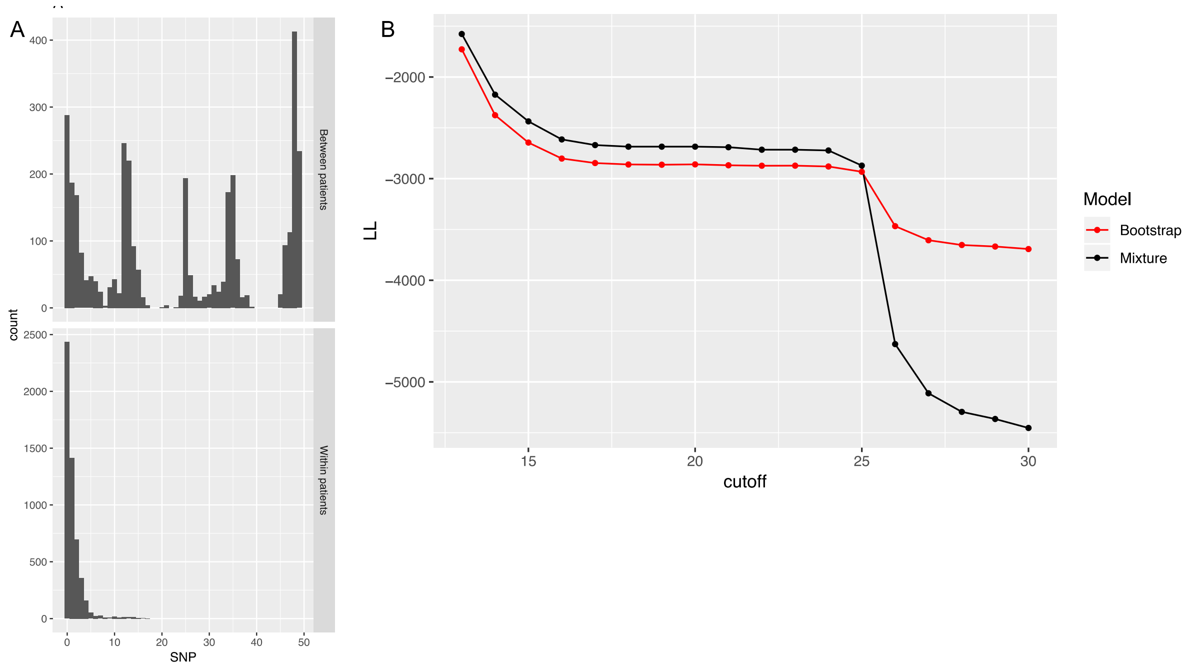

A: Distribution of SNPs (capped to 50) for pairs of isolates belonging to the same ST, either from different patients (top panel) or form the same patients (bottom panel). Mixtures of 2, 3 or 4 Poisson distributions were fitted to the within-patient SNPs by maximum likelihood; the mixture with the lowest Akaike Information Criterion (AIC) value was that with 3 components with means 0, 2.5 and 12.4 SNPs and respective weights 15%, 70% and 15%. We then fitted the same three Poisson components (with free weights) to subsets of the between-host pairs from 0 up to an arbitrary cut-off, ranging from 13 SNPs to 30 SNPs. The log-likelihood is shown as a black line in **B.** To determine an appropriate cut-off, we compared the likelihood values for the between-host SNPs ("mixture" black line) to the range of likelihood values that would be obtained by resampling 10,000 times the mixture model fitted to the within-host data ("bootstrapped likelihood") up to each given cut-off value (shown on the x-axis. The red line in (B) shows the bottom 1% of the bootstrapped log-likelihood for each cut-off value. For cut-off values up to 25 SNPs, the likelihood of the within-patient mixture model fitted to the between-patient data (black line) is greater than the bottom 1% of the bootstrapped likelihood for the within-patient SNPs, suggesting that the between-patient SNPs up to that cut-off could have been drawn from the same distribution as the within-patient SNPs. In contrast, the between-patient likelihood falls sharply below the within-patient bootstrapped likelihood range if we include pairs of isolates with more than 25 SNPs, suggesting that such pairs of between-patient isolates could not have been drawn from the same distribution as within-patient pairs.

Supplementary Figure 3. Transmission network of *E. coli* STs shared by patients based on 17 SNP cut-off

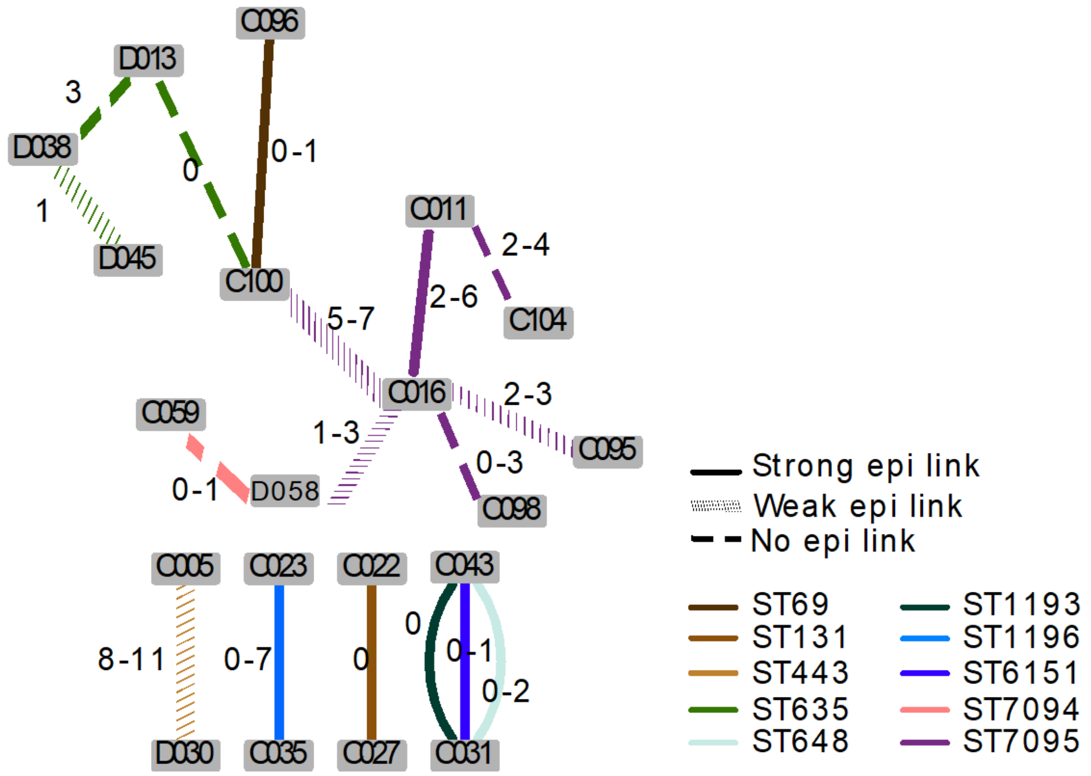

For each patient node, lines show the shortest genetic link to a previously sampled patient (putative transmission). Line colours show the ST transmitted and line types demonstrate the epidemiological link. Admission to the same bay, room or ward at the same time or within 7 days were classified as strong epidemiological links, while admissions in the same ward separated by more than 7 days or to the study hospital but to different wards were classified as weak epidemiological links and no epidemiological links was reported if neither of these occurred.

Supplementary Figure 4. ***E. coli*** **ST7095 transmission cluster, showing phylogenetic tree and epidemiology**

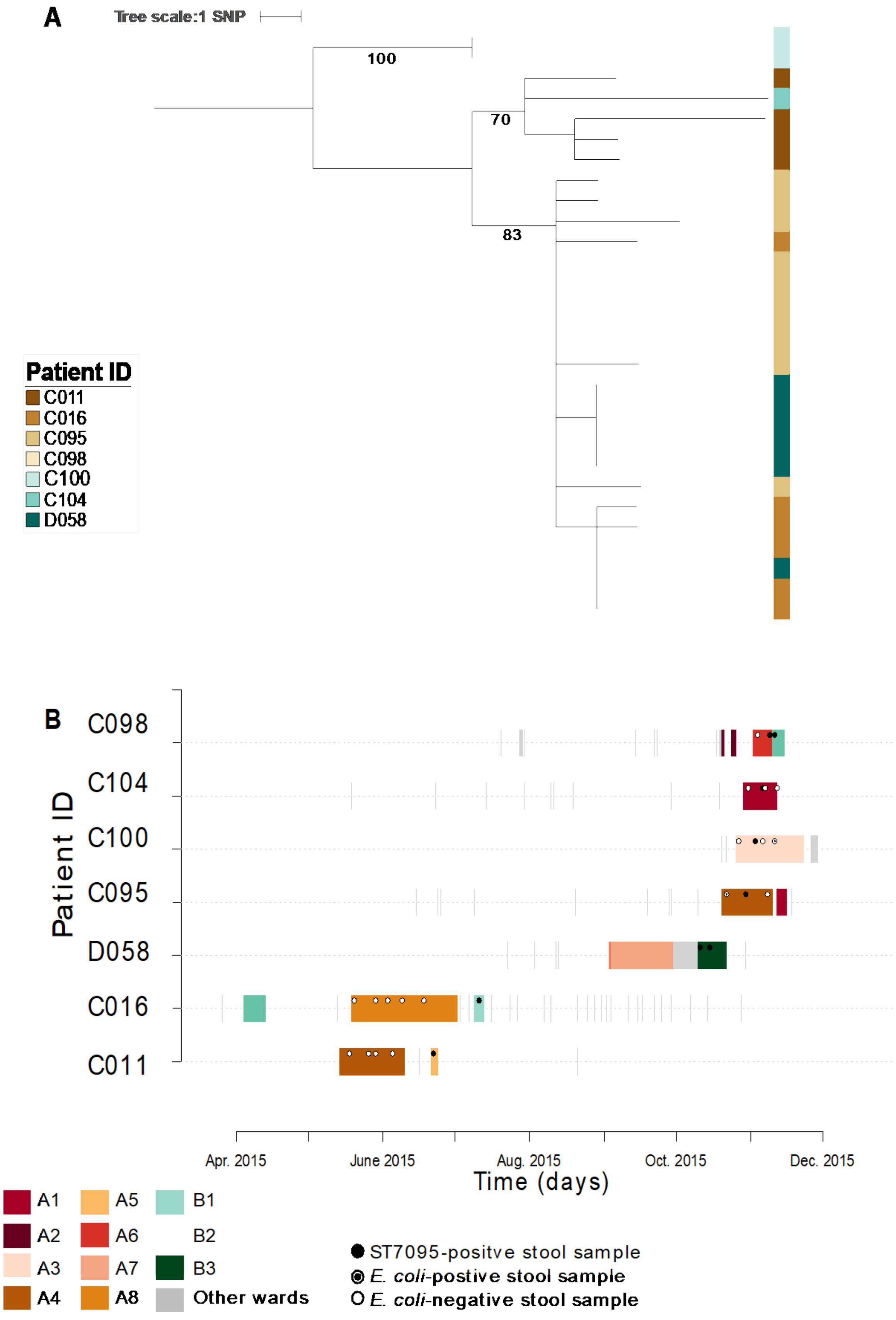

A: Maximum likelihood phylogenetic tree of 29 ST7095 *E. coli* isolates from 7 patients. Numbers below branches are bootstrap support values from 100 replicates if equal to or larger than 70%. **B:** Hospital admissions for the 7 ST7095 patients involved in the same transmission cluster are represented as rectangles. Visits at wards A and B are colour-coded by room (see legend), and visits to other wards are shown in grey. Positivity results for *E. coli* and ST7095 are shown as circles (see legend). All patients were linked by isolates that were within 17 SNPs. Negative *E. coli* screens prior to a positive *E. coli* screen for six patients (C098, C104, C100, C095, C016, and C011) further support hospital acquisition.

Supplementary Figure 5. ST635 transmission cluster, showing phylogenetic tree and epidemiology.

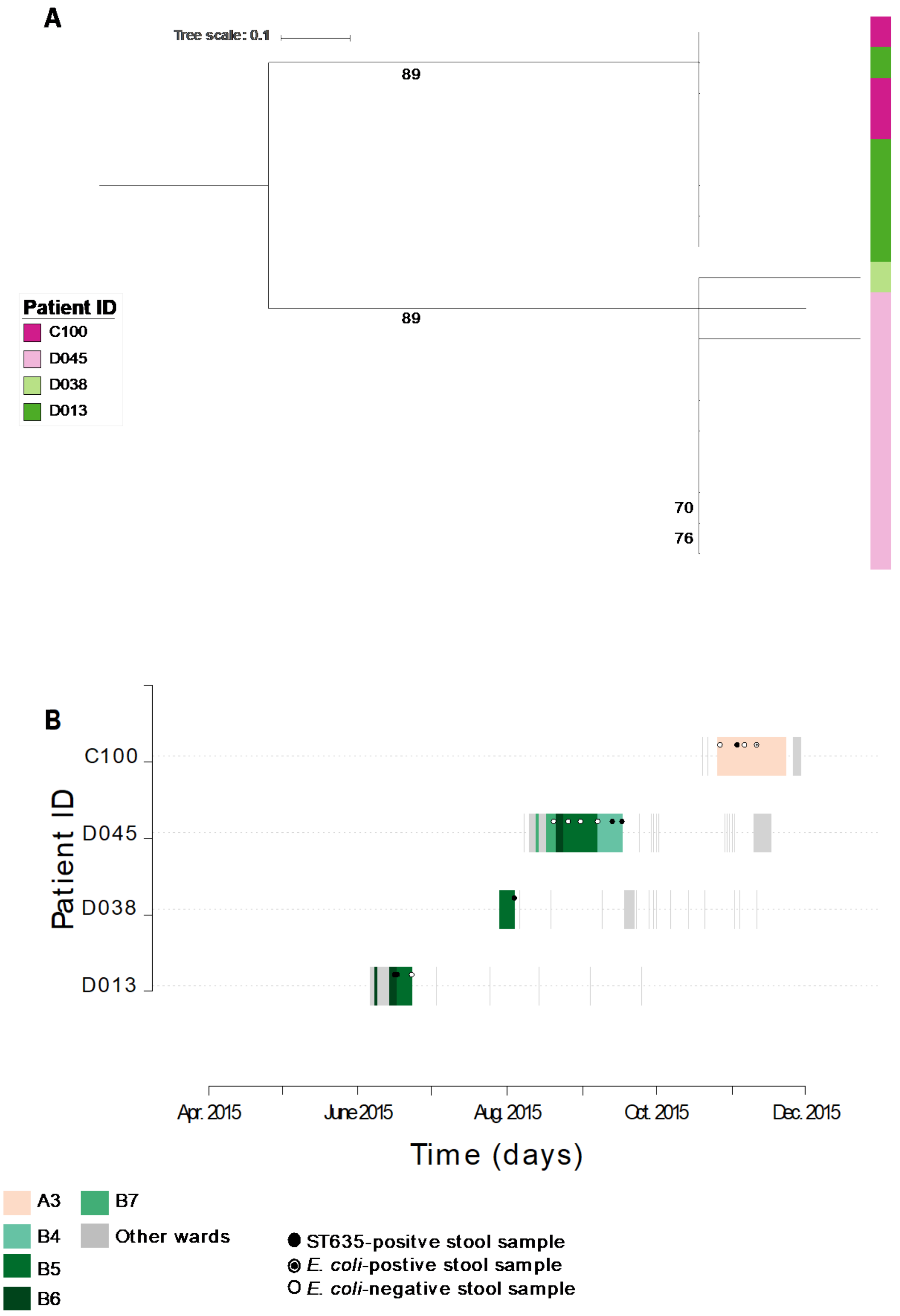

A: Maximum likelihood phylogenetic tree of 18 ST635 *E. coli* isolates from 4 patients. Numbers below branches are bootstrap support values from 100 replicates if equal to or larger than 70%. **B.** Hospital admissions for the 4 ST7095 patients involved in the same transmission cluster are represented as rectangles. Visits at wards A and B are colour-coded by room (see legend), and visits to other hospital wards are shown in grey. Positivity results for *E. coli* and ST635 are shown as circles (see legend). All patients were linked by isolates that were within 17 SNPs. Negative *E. coli* screens prior to a positive *E. coli* screen for six patients (C098, C104, C100, C095, C016, and C011) further support hospital acquisition

Supplementary Figure 6. Circular maps of representative plasmids carrying the *bla*$_{CTX-M15}$ or *bla*$_{CTX-M14}$ resistance genes.

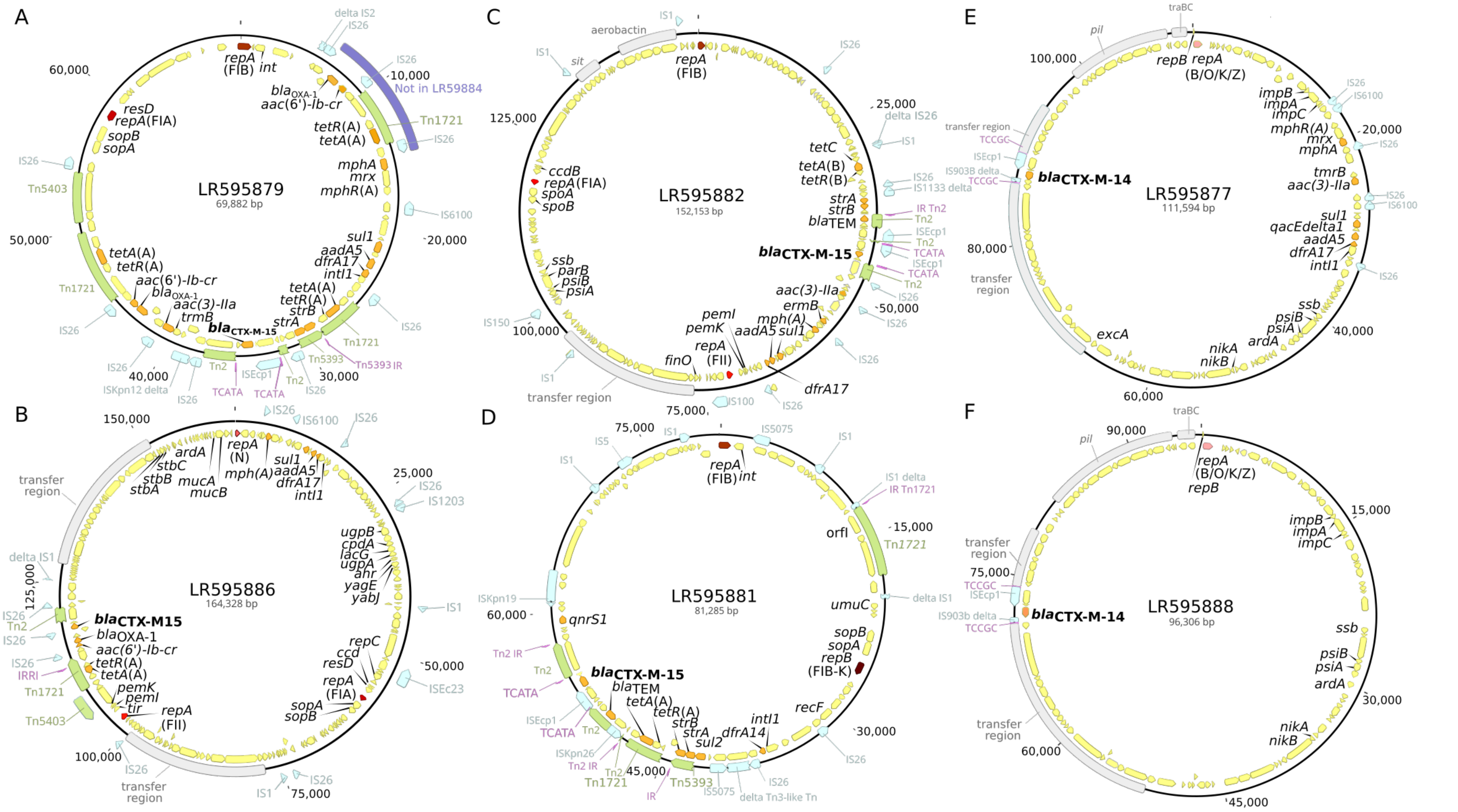

Yellow, orange and shades of red arrows represent open reading frames, with antibiotic resistance genes in orange and those encoding plasmid replication initiation proteins in red. Green boxes indicate the transposon derivation of specific regions and insertion sequences (IS) are shown in light blue with IS names indicated. Inverted and direct repeats are in pink and labelled by the transposon they are from or by the sequence repeated. Sets of genes involved in plasmid transfer, pilus assembly (*pil*) or virulence-associated-genes including aerobactin (siderophore system) and *sit* (iron and manganese ABC transport system) and indicated by grey boxes. A dark blue box in LR59879 indicate the extent of the region missing in LR59884. Geneious version 11.1 created by Biomatters was used in making this figure.

Supplementary Figure 7. Comparison of the B/O/K/Z plasmids isolated from patient C047

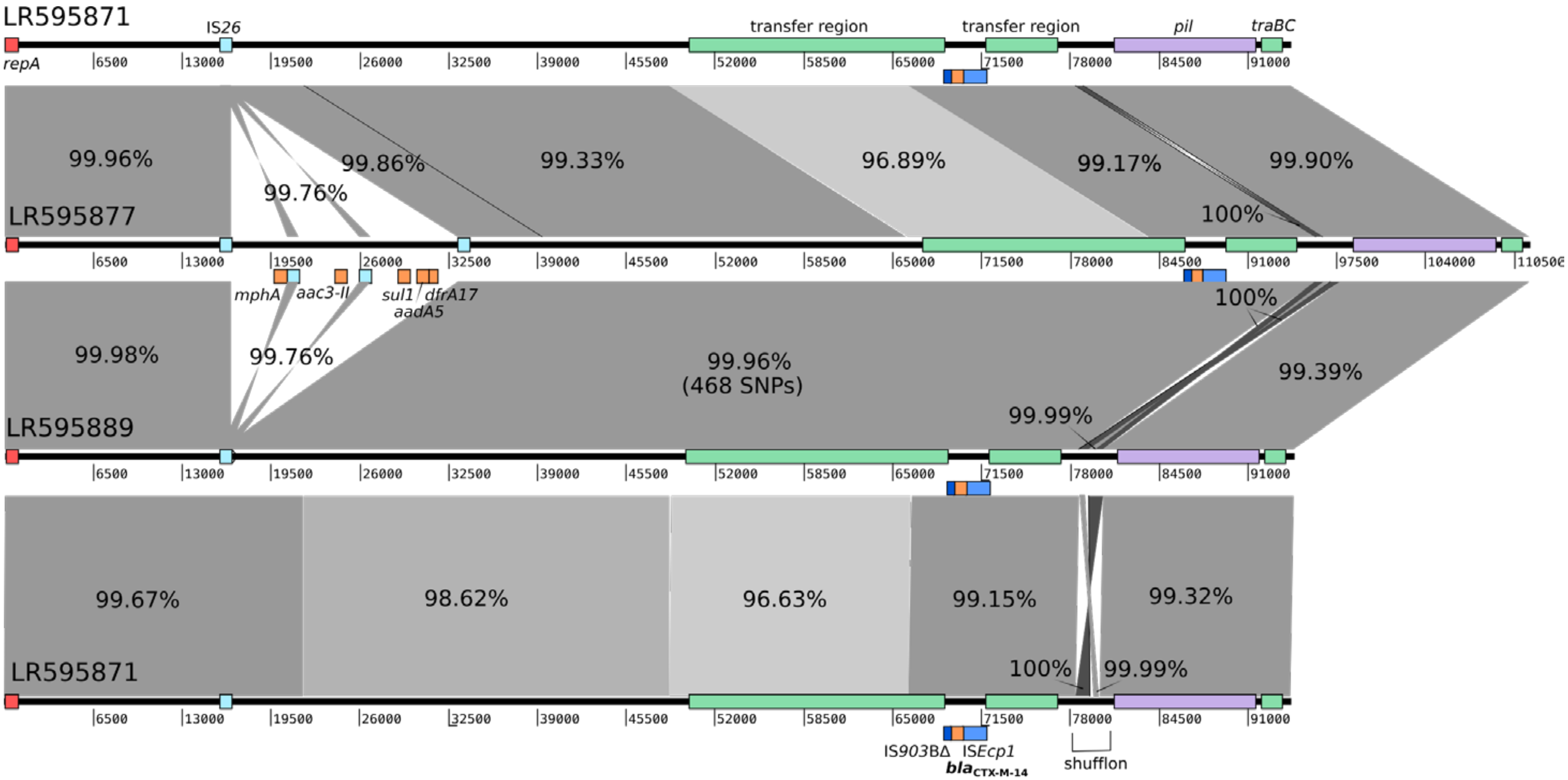

Regions of homology are shown by grey boxes and the numbers display DNA identity. Feature of the plasmid are indicated by coloured boxes, including the *repA* gene, sets of genes involved in plasmids transfer and pilus assembly, in red, green and purple respectively. Insertion sequences are in shades of blue and their IDity is indicated. Antibiotic resistance genes are shown in orange. ACT was used to visualise the BLAST comparison of the plasmids. These CTX-M-

14 plasmids (LR595889, LR595871, LR5R5877) had a sequence ID ranging from 98.6 – 98.9% and varied in size and plasmid composition. LR5R587 contained an IS*26*-bounded segment carrying additional resistance genes (orange).

5. SUPPLEMENTARY REFERENCES

References

1. Zerbino DR, Birney E. Velvet: algorithms for de novo short read assembly using de Bruijn graphs. *Genome Res* 2008; **18**.
2. Boetzer M, Henkel CV, Jansen HJ, Butler D, Pirovano W. Scaffolding pre-assembled contigs using SSPACE. *Bioinformatics* 2011; **27**(4): 578-9.
3. Boetzer M, Pirovano W. Toward almost closed genomes with GapFiller. *Genome Biology* 2012; **13**(6): R56.
4. Seemann T. Prokka: rapid prokaryotic genome annotation. *Bioinformatics* 2014; **30**(14): 2068-9.
5. Pruitt KD, Tatusova T, Brown GR, Maglott DR. NCBI Reference Sequences (RefSeq): current status, new features and genome annotation policy. *Nucleic Acids Res* 2012; **40**.
6. Page AJ, Cummins CA, Hunt M, et al. Roary: rapid large-scale prokaryote pan genome analysis. *Bioinformatics* 2015; **31**(22): 3691-3.
7. Stamatakis A. RAxML version 8: a tool for phylogenetic analysis and post-analysis of large phylogenies. *Bioinformatics* 2014; **30**(9): 1312-3.
8. Hunt M, Silva ND, Otto TD, Parkhill J, Keane JA, Harris SR. Circlator: automated circularization of genome assemblies using long sequencing reads. *Genome Biology* 2015; **16**(1): 294.
9. Croucher NJ, Page AJ, Connor TR, et al. Rapid phylogenetic analysis of large samples of recombinant bacterial whole genome sequences using Gubbins. *Nucleic Acids Res* 2015; **43**.
10. Johnson JR, Tchesnokova V, Johnston B, et al. Abrupt emergence of a single dominant multidrug-resistant strain of *Escherichia coli*. *The Journal of Infectious Diseases* 2013; **207**(6): 919-28.
11. Sánchez-Céspedes J, Sáez-López E, Frimodt-Møller N, Vila J, Soto SM. Effects of a mutation in the *gyrA* gene on the virulence of uropathogenic *Escherichia coli*. *Antimicrobial Agents and Chemotherapy* 2015; **59**(8): 4662-8.
12. Heisig P. Genetic evidence for a role of parC mutations in development of high-level fluoroquinolone resistance in *Escherichia coli*. *Antimicrobial Agents and Chemotherapy* 1996; **40**(4): 879-85.
13. Hunt M, Mather AE, Sánchez-Busó L, et al. ARIBA: rapid antimicrobial resistance genotyping directly from sequencing reads. *Microbial genomics* 2017; **3**(10): e000131-e.
14. Falgenhauer L, Yao Y, Fritzenwanker M, Schmiedel J, Imirzalioglu C, Chakraborty T. Complete genome sequence of phage-like plasmid pECOH89, encoding CTX-M-15. *Genome Announc* 2014; **2**(2): e00356-14.
